# A global database of historic and real-time flood events based on social media

**DOI:** 10.1038/s41597-019-0326-9

**Published:** 2019-12-09

**Authors:** Jens A. de Bruijn, Hans de Moel, Brenden Jongman, Marleen C. de Ruiter, Jurjen Wagemaker, Jeroen C. J. H. Aerts

**Affiliations:** 1 Institute for Environmental Studies, VU University, De Boelelaan 1087, 1081HV Amsterdam, The Netherlands; 2 World Bank Group, Washington D.C., 20433 USA; 3 FloodTags, Binckhorstlaan 36, The Hague, 2511 BE The Netherlands

**Keywords:** Natural hazards, Geography, Hydrology

## Abstract

Early event detection and response can significantly reduce the societal impact of floods. Currently, early warning systems rely on gauges, radar data, models and informal local sources. However, the scope and reliability of these systems are limited. Recently, the use of social media for detecting disasters has shown promising results, especially for earthquakes. Here, we present a new database for detecting floods in real-time on a global scale using Twitter. The method was developed using 88 million tweets, from which we derived over 10,000 flood events (i.e., flooding occurring in a country or first order administrative subdivision) across 176 countries in 11 languages in just over four years. Using strict parameters, validation shows that approximately 90% of the events were correctly detected. In countries where the first official language is included, our algorithm detected 63% of events in NatCatSERVICE disaster database at admin 1 level. Moreover, a large number of flood events not included in NatCatSERVICE were detected. All results are publicly available on www.globalfloodmonitor.org.

## Abstract (summary)

**Table:** 

Measurement(s)	Internet Social Media • geographic location • flood
Technology Type(s)	digital curation • computational modeling technique
Factor Type(s)	text • timestamp
Sample Characteristic - Environment	flood
Sample Characteristic - Location	Earth (planet)

Machine-accessible metadata file describing the reported data: 10.6084/m9.figshare.11294915

## Background & Summary

Each year, flooding causes major damages and affects millions of people around the globe^1^. For flood detection we traditionally rely on gauge systems in rivers, remote sensing information^2^, and other sources such as reports from the media and governmental agencies^3^. Yet, a large number of flood events remain unreported^4^. Moreover, the data recorded in traditional databases is often acquired through manual observation, and processing and publication can take days, weeks, or even months. In addition, the results of existing databases, especially from the (re-)insurance sector, are not always freely accessible.

Over the last decade, social media platforms such as Twitter have emerged as highly effective sources of information that facilitate rapid collection and spreading of news from local sources, effectively using humans as a sensor^5^, increasing situational awareness of otherwise not or late-detected events^3,6^. Therefore, the real-time analysis of social media data enables early event detection and has become a commonly used data source due do the volume and availability through a streaming API^7^. However, the analysis of Twitter data is challenging because, for example, Twitter users are unevenly distributed across the globe, are more active during the day than at night and their tweets are often ungrammatical, error-prone and multilingual^8^. Nevertheless, significant advances have been made in the automated analysis of tweets^9,10^ for detecting natural hazards and other events^5,6,11,12^.

The vast majority of specified event detection studies using Twitter have focused on the detection of earthquakes^5,11^. However, the differences in hazard characteristics significantly influence the possible approaches in detecting flood events at a global scale. Earthquakes are a sudden onset event of which, on a global scale, generally no major simultaneous events occur. Most floods have a relatively slow onset (compared to earthquakes) and often gradually inundate an area. They can occur almost anywhere and on a global scale there are commonly multiple simultaneous events^13^. Moreover, some of these events attract much more attention globally than others. For example, when Hurricane Harvey hit Houston, hundreds of thousands of flood-related messages were posted globally, thus also resulting in a burst of flood-related tweets all around the world. Concurrently to Hurricane Harvey, flood events were ongoing in data-scarce countries such as Nigeria and Uganda mentioned in only a few tweets. For the global monitoring and data storing of flood events the challenge thus comprises the monitoring of multiple simultaneous heterogeneous events that can have a slow or sudden onset across different languages and world regions in both data-rich and data-scarce environments. A solution is to first employ geoparsing on the tweet’s text and then perform event detection. The geoparsing process is used to recognize and disambiguate location mentions within the tweets’ text^14–18^.

Therefore, we designed a method for locating and detecting flood events on a global scale and present the resulting real-time and historic database. The historic database of events is based on ~88 million tweets collected over 4 years in 12 languages. The method used to derive this database works in real-time, on a global scale in both data-rich and data-poor regions. After collecting and filtering tweets, we employ the “Toponym-based Algorithm for Grouped Geoparsing of Social media” (TAGGS), a multi-lingual global geoparsing algorithm, to extract mentions of location from the content of tweets^14^. Subsequently, these locations are used to detect sudden bursts in the number of flood-related tweets linked to countries and their first order administrative subdivisions (admin 1), such as provinces and states. Here, we follow the admin 1 subdivisions as defined in the GeoNames database (www.geonames.org), except for the United Kingdom and Sri Lanka where the second order administrative subdivision (admin 2) is used (i.e., counties and districts). In this work, we define such a burst in the number of flood-related tweets within a region (i.e., country or admin 1) as a flood event. In addition, we developed a freely accessible web platform, where researchers and disaster managers can view flood events in real-time and access historically detected floods. We validated our flood events database by comparing our results to events registered in Munich Re’s NatCatSERVICE (natcatservice.munichre.com) and by manually verifying non-registered events.

The historic and real-time database can be used to, for example, (1) validate flood risk models, (2) use real-time information for data assimilation in flood prediction models, (3) task satellites allowing for the collection of remote sensing data of individual events^19^, (4) improve early warning systems and situational awareness to reduce the impact of extreme floods^20^ and (5) improve applications that depend on historical information, such as forecast-based financing schemes^21^.

## Methods

This section first discusses existing methods that use Twitter for event detection. Next, it describes the processes of collecting and filtering Twitter data, the extraction of location mentions from these tweets and their assignment to regions. Finally, we describe how events are detected within these regions.

### Review of other methods

Atefeh *et al*.^9^, present a comprehensive overview of different methods and challenges for the detection of events using Twitter. Here, we discuss the relevant methods and pitfalls in using Twitter for global flood event detection. Twitter has extensively been used for earthquake event detection.

Most research performs event detection first, sometimes followed by localization. For example, Sakaki *et al*.^22^ describe a system that collects tweets, filters them using a support vector machine. They then detect events using a system that estimates the probability of a certain number of sensors reporting an event within a certain time interval, assuming the time that users take to write a tweet follows an exponential distribution. Once an event is detected, localization of the event is done using Kalman and particle filters based on the GPS-coordinates of the tweets and their users’ registered locations. In another work, Avvenuti *et al*.^5^ describe a Twitter-based system that monitors earthquakes in Italy. First, tweets with a series of related keywords are collected, which are then filtered by a series of text classifiers. Then, the system checks how much higher the frequency is of related messages of a (current) fixed-length short-term time window compared to a long-term time window. If this ratio is higher than a certain threshold an event is recorded. Once the event is detected, a module extracts mentioned locations and GPS-coordinates to obtain reports of damages. Poblete *et al*.^11^ note that, in most works, irrelevant messages are discarded beforehand using ad hoc filters or classifiers. They propose a system that considers the number of relevant *data elements* (e.g., a reference to an earthquake event) relative to the total number of messages within fixed time windows. Once an event is detected, it is assigned to a certain country by finding the most-mentioned country within the relevant tweets. However, approaches where localization is performed afterwards are not feasible for floods, as there are often multiple floods ongoing at the same time.

In some other works, localization is performed first, followed by a detection step^7,23^. For example, Sarmiento *et al*.^23^ propose a system that first finds location mentions from the tweet’s text and then detects events based on anomalies in the activity related to geo-locations within fixed time windows. For floods, Arthur *et al*.^7^ detect areas with ongoing flooding on a relatively high-detail in the data-rich UK. In their system, locations are first assigned to tweets based on GPS-locations, places mentioned in the tweet’s text and user hometowns. Then enhanced Twitter activity is detected in certain raster cells using tweets collected over a fixed time period. However, the study focusses only on relative floodiness (i.e., some regions are more likely to be flooded at this time than others). While this is useful for some use cases, such as forecast verification, absolute flooding is not detected. Jongman *et al*.^3^ show that an increase in geoparsed tweets occurs during flood events, which in some cases, allows for faster flood detection than using other traditional methods. However, no event detection step is employed. Rossi *et al*.^6^ detect flood events in northern Italy using flood-related tweets written in Italian using the Extreme Studentized Deviate test using fixed time windows. However, we argue that it is problematic to use fixed-length time windows, especially in data-poor environments. Due to the lack of data, models would need to use a long time window in these areas to detect slow-onset or mild events. However, this also means that the detection of a rapid onset severe event takes much longer. In contrast, a flexible time window algorithm can detect events at the moment sufficient data is available irrelevant of a fixed time window.

In conclusion, to the best of our knowledge, no algorithm has been developed that can detect many simultaneous flood events from social media on a global scale. Moreover, previous algorithms were not used to construct a multi-year database and systematically validated.

### Data collection

To create the Global Flood event database and ongoing monitoring of events, Tweets are collected in 12 major languages (Table 1) with the Twitter real-time streaming (API) using pre-selected flood-related keywords between July 30, 2014 and November 20, 2018, and across a global scale. These tweets mentioned one or more flood-related keywords in one of 11 major languages (Table 1).

**Table 1:** Keywords related to floods used for analysis.

Language	Keywords
English	flood, floods, flooding, flooded, inundation, inundations, inundated
Indonesian	banjir
Filipino	baha, bumabaha, pagbaha
French	inonder, inondation
German	flut, hochwasser, Überflutung
Italian	inondazione, alluvione
Dutch	overstroming
Polish	powódź, powodzie
Portuguese	inundação, inundacão, inundaçao, inundacao, inundações
Spanish	inundación, inundacion, inundar, inundaciones
Turkish	su taşkın, su baskını, sel bastı, sel suyu, sel yüzünden, taşkın oldu, sel suyunun

### Location extraction and assignment to regions

To extract locations from tweets we use the process of “geoparsing”^14,24^. Here we employ the TAGGS algorithm^14^. The algorithm does not assume a-priori knowledge about an event and works on a global scale in space-separated languages. Using this algorithm we extract countries, administrative areas, and settlements (i.e., cities, towns and villages) that were mentioned within the tweets’ text. This process consisted of two steps: (1) toponym recognition, and (2) toponym resolution. In the toponym recognition stage, the text of each tweet is split into individual words, a process also referred to as “tokenization”. Next, these tokens, such as “Amsterdam”, as well as consecutive tokens, such as “New York,” are matched to a near-comprehensive set of geographical locations extracted from the GeoNames database, as well as OpenStreetMap (www.openstreetmap.com) for Myanmar. This process yields a list of toponyms mentioned in the text of tweets with their candidate locations. For instance, the toponym “Boston” yields among others “Boston, USA,” “Boston, UK.” In the toponym resolution step, a score is assigned to each of these candidates by matching them with other spatial indicators available in the tweet, i.e., tweet coordinates, user time zones, user hometowns and mentions of nearby locations. Then, tweets that mention the same toponym within a 24-hour time frame are grouped, which allows us to calculate an average score for each candidate location. Finally, the location with the highest score is selected and assigned to all tweets in the group. TAGGS has been extensively validated on global flood-related tweets for unspecified events (precision, recall, F_1_-score: 0.92, 0.84, 0.88).

Precision (equation 1) is the fraction of the number of correctly classified relevant instances (i.e., true positives) among the total number of instances classified as relevant (i.e., true positives and false positives). Recall (equation 2) is the fraction of the correctly classified relevant instances (i.e., true positives) among all relevant instances (i.e., true positives and false negatives). The F_1_-score is the harmonic mean of precision and recall.

1 $$precision=\frac{true\,postive}{true\,position+false\,position}$$

2 $$\begin{array}{c}Recall=\frac{true\,positive}{true\,positive+false\,negative}\end{array}$$

Moreover, TAGGS shows a clear relationship between the number of tweets in a country and the damages due to flooding events. A full explanation and validation of this process can be found in^14^.

Based on the location(s) found, each tweet was then assigned to a single region, to multiple regions, or to no region at all. Tweets that mentioned a country were assigned to the region representing that country, and tweets that mentioned a settlement within an admin 1 subdivision or the subdivision itself were assigned to the respective subdivision. The flood detection algorithm aims to detect flood events at the levels of both country and their admin 1 subdivisions by detecting changes in the number of flood-related tweets for these regions. We note here that the administrative level at which floods are detected is, in fact, arbitrary and could easily be adapted to any administrative level. We find that in areas with a high internet penetration, a large number of people refer to the admin 1 level (e.g., “*From Florida to Georgia Irma causes heavy #floods*”), while in areas where internet penetration is much lower, a flood is frequently only referred to at the country level (e.g., “*Floods - Mozambique Flood (Severe)*”). Therefore, given the global scope of this paper, we perform detection at the aforementioned administrative levels.

### Filtering

Unfortunately, the word “flood” and its translations are often used figuratively (e.g., “*a flood of joy*”) or in transferred sense (e.g., “*a flood of tears*”) in many languages. Moreover, the tweets do not always refer to currently ongoing flood events but can refer to historic or future (forecasted) events. To filter the tweets, we can use algorithms from the field of natural language processing^9^. Recently, large advances have been made in the (multi-lingual) classification of text^25–27^.

Here, we adopt the cased multilingual version of BERT (Bidirectional Encoder Representations from Transformers) to classify tweets in 2 categories^26^: related to an ongoing event (i.e., “relevant”) and not related to ongoing event (i.e., “irrelevant”). BERT is a deep learning-based natural language processing (NLP) model that learns relations between words and sub-words in a text (i.e., word embeddings) and uses these to encode text, followed by a decoder to make predictions. We obtain the pre-trained version of BERT which can then be fine-tuned for a specific task. To this extent, we first manually label 100 tweets that have a location attached in the localization extraction step for each language. For the three most abundant languages (i.e., English, Indonesian and Spanish) we label an additional 100 tweets. To obtain tweets that are both spatially and temporally varied, we obtain these tweets using the following procedure for each language:

1. Select a random day on which at least one tweet was sent in a particular language.
2. Select a random region (i.e., country or admin1) that has been tweeted about on the selected day.
3. Select a random tweet that was tweeted on the selected day about the selected region.
4. Check if the tweet is still available from the Twitter API and if all the links can still be resolved and the linked content is available (i.e., the article is still available, no paywall etc.)
5. Repeat the previous steps from the start.

In this labelled dataset of 1400 tweets, 44% of tweets is labelled as relevant. Then, we design a classification algorithm based on BERT. This pre-trained model feeds into a dropout layer used for training (dropout rate is 0.5) and a dense fully connected layer with a single sigmoid output node. Subsequently, we train the model using the rectified Adam optimizer^28^ with a learning rate of 3 × 10^−5^ and warmup proportion of 0.1 over 6 epochs using 80% of the labelled tweets as training data and the other 20% as validation data. Moreover, we up-weigh the contribution of the “relevant” class inversely proportional to the frequency of the “relevant” labels in the training data, balancing the contributions of the “relevant” and the “irrelevant” labels.

The resulting model has a precision, recall and F_1_-score of respectively 0.77, 0.83, and 0.80. Finally, the fine-tuned model is then applied to all tweets where a location was assigned, discarding all irrelevant tweets.

As an additional filtering step, we discard (near-) duplicate tweets. Here we assume that a single tweet is unreliable, but a large number of unreliable tweets mentioning a flood in the same region is reliable. However, this is only true if the content of the tweets is based on independent observations. This is an assumption that does not hold for Twitter, because people are likely to copy or modify another tweet or are likely to tweet about similar online or offline content, such as the header of a news article^29^. Therefore, to sanitize the tweet database, we discard (1) retweets, (2) tweets from users that already posted a flood-related tweet in the last 14 days about a specific region and (3) tweets that were exact or near copies of previous tweets (e.g., news articles tweeted by different persons using the same headline) within the same region. A tweet was considered a (near-) copy if more than five consecutive words matched with those in another tweet among the previous 100 tweets assigned to a region. If a tweet contained less than five words in total, only those words were used to find exact or near copies.

### Burst detection

Finally, in order to detect events from online media, a burst detection step is required. Several approaches have been developed for detecting enhanced Twitter activity. These approaches include: infinite state automatons^30^, a comparison of the number of keywords in consecutive time windows^11,31,32^, Bayesian change point detection^33^, and sequentially checking whether the time between consecutive data points is shorter than a certain time threshold (SEQAVG)^34^. For this study, we employed the SEQAVG method to detect bursts because the algorithm does not require a fixed time window but can rather detect a burst on each incoming tweet rather than at the end of a fixed time window. As such it works both for relatively sudden events and events that develop in the course of hours or days. Moreover, it can be adapted to work in data-rich and data-sparse areas and is computationally efficient. We expanded the algorithm in two ways: (1) by accounting for the fluctuations in numbers of Twitter users according to time of day^8^ and (2) by employing an adaptable threshold for the detection of bursts.

A region (i.e., country or admin 1 subdivision) can be either in a “flooded” state with enhanced Twitter activity, or a normal state. It was assumed that each region started in the normal state. Because some regions were likely to be experiencing a flood event during at the first day of that Twitter data was collected, we excluded the first 30 days of model output from the analysis.

In short, the algorithm keeps track of the time passed between consecutive tweets assigned to each region. By analysing the time differences between these tweets and comparing them to a region-specific threshold, the state of the region was set to the flood state during enhanced Twitter activity and to the normal state when the Twitter activity reverted to normal levels of activity.

#### Day/night variations

Each tweet was analysed further by calculating the time elapsed since the previous tweet (Δ*t*). To correct for the variation between daytime and night-time Twitter activity, an hourly correction factor was applied (*c*_*h*_). Using over three years of geoparsed tweets^14^, the number of hourly tweets assigned to each region (*n*_*h*_) was determined. Using equation 3, a centred moving average ${\bar{n}}_{h}$was calculated using two data points on either side of the central value:

3 $$\begin{array}{c}{\bar{n}}_{h}=\frac{{n}_{h-2}+{n}_{h-1}+{n}_{h}+{n}_{h+1}+{n}_{h+2}}{5}\end{array}$$

Next, using equation 4 the sum of the values was normalized to one:

4 $$\begin{array}{c}{c}_{h}=\frac{{\bar{n}}_{h}}{{\sum }_{h=1}^{24}{\bar{n}}_{h}}\,\ast \,24\end{array}$$

Finally, using equation 5, the corrected time (Δ*t*_*c*_) was calculated between tweets by applying the hourly correction factor:

5 $$\begin{array}{c}\Delta {t}_{c}=\Delta t\,\ast \,{c}_{h}\end{array}$$

If there were less than 100 tweets assigned to a region over the three years of collected data for an admin 1 subdivision, we calculated the correction factor at the country level. If the number of tweets was less than 100 for an entire country, no correction factor was applied.

#### Event detection

Even when no flood was taking place, some tweets mentioning floods were expected. Some of these related to other minor issues (e.g., “tea flooding the floor because a cup fell off”) and others related to historic flood events (e.g., “today we commemorate the devastating floods of 1953”). This volume of tweets varied both temporally and spatially. For example, the average number of flood-related tweets about Jakarta was much higher than that of other flood-prone regions with much lower Twitter penetration, such as Mozambique. Therefore, to detect bursts of tweets, the average time between consecutive tweets was compared to a region-specific threshold for the start of an event (θ_s_) and the end of an event (θ_e_). If the corrected time between tweets was shorter than threshold θ_s_, an event was suspected. We employed different thresholds for the start and end of an event as we find, in general, a relatively high number of tweets during the start of the event followed by a relatively gradual decay over time as the event further develops. Next, depending on the region’s expected time between tweets, more tweets were accumulated in a local array with length *n* according to the following equation 6, where θ_s_ is specified in days and *c* is a constant:

6 $$\begin{array}{c}n=7\,* \,{e}^{-{{\rm{\theta }}}_{s}}+c\end{array}$$

The formula was designed so that the size of the array is larger for regions with a short time between tweets, i.e., regions with a high tweet volume, than for regions with a long time between. Varying the parameter *c* affects the sensitivity of the algorithm. When using a low value for *c*, the model is expected to be relatively sensitive, whereas high *c* decreases sensitivity. A high sensitivity setting results in earlier and more frequent event detection, which could benefit applications such as disaster response. Although this setting is also more likely to yield false positive events, the event can be easily verified manually by reading the tweets, before deciding to act. By contrast, a low sensitivity setting could be used to create a relatively reliable event database. To test various settings, we performed three model runs, using *c* = *1*, *c* = *2* and *c* = *3* for a *sensitive*, *balanced* and *strict* run respectively.

**Fig. 1:**
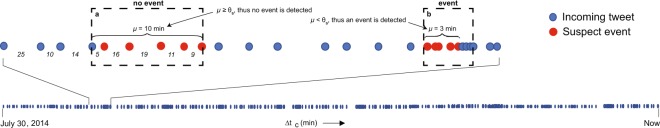
Schematic example of the event detection algorithm. “a” and “b” show two time intervals where an event is suspected. At “a” the event is not confirmed because the average corrected time is above the threshold. At “b” the event is confirmed.

If the average corrected time between the tweets in a local array is also lower than the threshold θ_s_, the region is set to the flood state. Figure 1 shows an example of corrected time intervals between tweets for a region with two suspected events: “a” and “b”. In this example, the threshold θ_s_ is set to 6 minutes. At “a,” the corrected time between two consecutive tweets is 5 minutes, which falls below the threshold, so an event is suspected. However, when accumulating several tweets in the local array, the average corrected time between tweets does not fall below the threshold, so the event is not confirmed. Later, at “b,” a new event is suspected. Now the average corrected time between tweets in the local array falls below the threshold, confirming the event and setting the region to the flood state. While the region is in the flood state, each new tweet is received and accumulated in the local array while the earliest tweet in the array is discarded. If the average corrected time between the accumulated tweets in the array falls above a threshold for the end of an event (θ_e_), or if no tweet was received over the last 24 hours, the region is set back to the normal state.

For the threshold θ_s_ and θ_e_, we respectively used 5% and 30% of the average expected time between consecutive tweets when no flood was occurring. To accurately determine these values, we use a spin-up period of 365 days. A period of 365 days was chosen to consider the (regional) popularity of Twitter at the time, while making sure the threshold is not heavily influenced by seasonality. At the start of the spin-up period the thresholds are set using the average expected time interval between tweets posted during the whole spin-up period, ideally, not considering tweets posted during flood events or their aftermath. Unfortunately, no a-priori knowledge was available about the timing of flood events, while many regions do experience flood events. To correct for this, we do not consider the time differences between tweets posted on the days with the 30% highest number of tweets. While the algorithm analysed tweets posted during the spin-up period (i.e., the first 365 days), the initial threshold was gradually replaced by an adaptive threshold. This adaptive threshold is based on only the tweets that were posted when the region was in the normal state (i.e., no flood was ongoing) during the last 365 days, allowing for better reflection of the actual tweet volume within an area when no event was ongoing. After the first 365 days of tweets were analysed, the initial threshold was completely replaced by the adaptive threshold and was continuously updated based on the intervals between tweets posted over the last 365 days, accommodating for (regional) changes in Twitter’s popularity.

Where a burst was detected in a region, that region was set to the flood state. The first tweet that signalled a suspect burst was labelled as the *start time* of the event. For most events, the burst was confirmed a few tweets after the first tweet (Fig. 1). This moment is labelled as the *time of detection*. The *end time* of an event was signalled when a region that was a flood state reverted to the normal state. When a new flood was detected within three days of the last flood in that same region, this event was considered a continuation of the previous event.

## Data Records

The detected flood events associated with this manuscript are available on Zenodo^35^. The repository contains three comma-separated values (CSV) files (Table 2), each for one of the sensitivity settings described (sensitive, balanced and strict). Moreover, a visualization of the recorded and real-time events is available on www.globalfloodmonitor.org, based on the latest version of the detection algorithm. A similar event table, which includes the real-time events, is available under the download-button. Researchers can also obtain access to the input data consisting of 88 million Tweet IDs for the purpose of non-commercial research and agree to Twitter Terms of Service, Privacy Policy, Developer Agreement, and Developer Policy. This data is available through Harvard Dataverse^36^ (version 2). From these, the full tweets that have not been deleted or otherwise made unavailable can be obtained using the Twitter API.

**Table 2:** The variable and their units for the detected events.

Entry	Variable name	Unit	Notes on variable
1	Event ID	—	Unique ID for event
2	Location ID	—	Location ID for the location in the GeoNames or OpenStreetMap database
3	Location name	—	Location name for the location in the GeoNames or OpenStreetMap database
4	Location URL	—	URL to the location in the GeoNames or OpenStreetMap database
5	Location type	—	Country, First order administrative subdivision
6	ISO 3116 country code	—	Three letter country code
7	Start time	Time	Start date and time of the event
8	End time	Time	End date and time of the event
9	Time of detection	Time	Date and time of the detection of the event

## Technical Validation

**Fig. 2:**
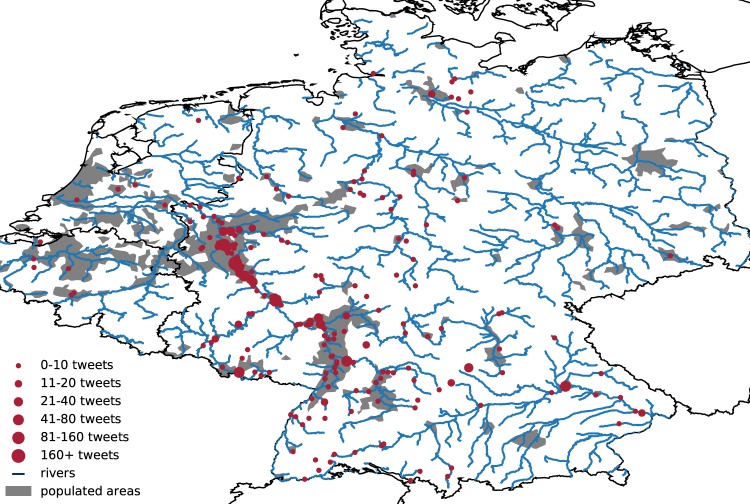
Tweets during the January 2018 floods in Germany, the Netherlands and Belgium. The tweets filtered tweets mentioning a settlement are shown in red overlaying populated areas (in grey) and the river network (in blue). Note that tweets that mention the country (i.e., Germany) or admin1 regions (i.e., states) are not shown here.

Out of the 87.6 million tweets mentioning at least one flood-related keyword, 30.9 million remain after localization and 11.5 million tweets classified as flood related remained after filtering using the BERT classification algorithm. As an example of these tweets at the level of settlements, Fig. 2 shows the river network of Germany, the Netherlands and Belgium overlaid by the mentioned locations during the floods in West Germany in January 2018. This shows that the located tweets are concentrated mainly in populated areas along the affected Rhine river. These tweets located at the level of settlements are later assigned to admin1 regions (Section 2.3). The tweets that mention the country (i.e., Germany) or admin1 regions (i.e., states) are not shown here.

Using the detection algorithm, we found a total 13,942, 10,561 and 8,588 flood events after the spin-up period (between July 30, 2015 and November 20, 2018) using the sensitive, balanced and strict run respectively (Table 3). For further validation, we only consider events that start within this period, unless stated otherwise. It should also be noted that when a flood event affects more than one administrative area or country, it can also be detected in multiple administrative areas and scales. This implies that large flood events can be accounted for multiple times by our algorithm.

**Table 3:** Number of events detected at the level of countries and first order administrative subdivisions for each sensitivity setting.

	country	admin1
Sensitive	2,554	11,388
Balanced	1,907	8,654
Strict	1,545	7,043

**Fig. 3:**
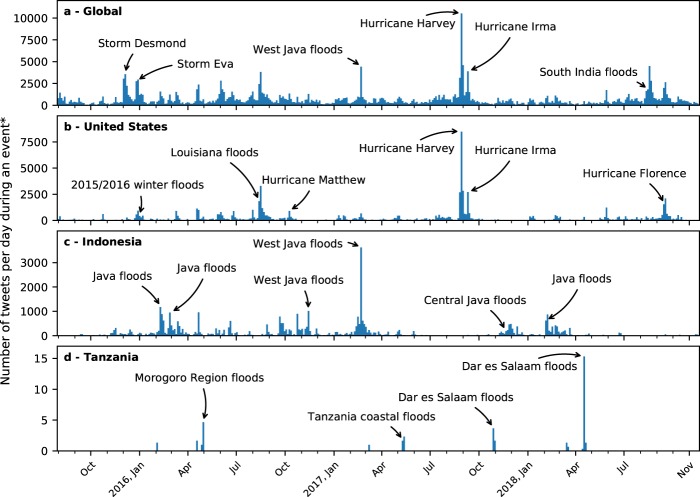
Detected events and number of “relevant” tweets. The panels show detected events globally (**a**), in the United States (**b**), in Indonesia (**c**) and in Tanzania (**d**). Events in admin1 regions are included. This figure only shows tweets (in blue) when part of a detected event. A selection of events is labelled for reference. *The number of tweets is displayed as a 3-day average as otherwise the bars become too small to be visible in the figure.

Figure 3 shows a timeline of these events and the number of non-duplicate tweets aggregated per three days within these events, at a global scale (Fig. 3a), for the United States (Fig. 3b), for Indonesia (Fig. 3c) and for Tanzania (Fig. 3d). The flooding in the United States clearly shows several hurricanes and tropical storms that hit its coastlines, as well as other riverine and flash floods. The flood patterns in Indonesia are related to the wet season, which runs from November to March. The exceptionally wet weather in Indonesia during the dry season, which runs from June to October of 2016, may be linked to La Niña conditions^37^.

### Validation with reported events

**Fig. 4:**
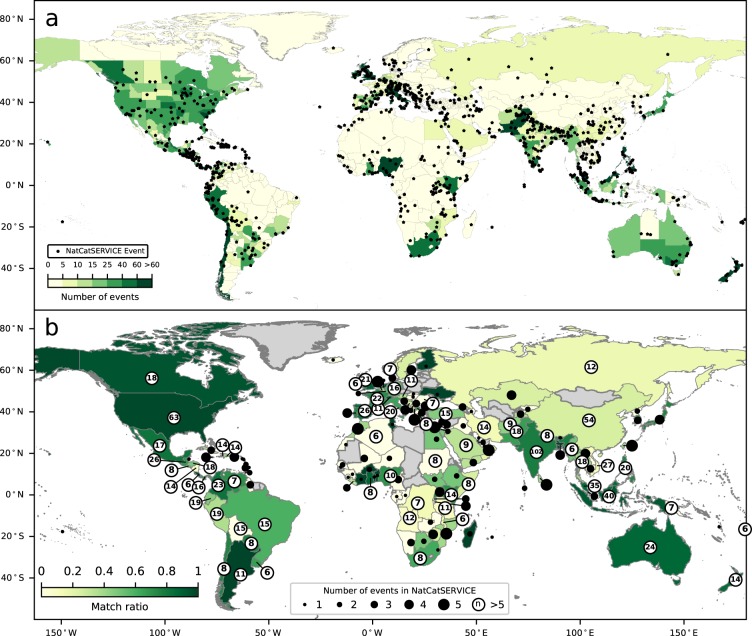
Comparison of detected events and NatCatSERVICE. Panel a shows the number of events detected by our algorithm at admin 1 level, either aggregated by country or shown at admin 1 level (yellow to green) and flood events in Munich RE’s NatCatSERVICE (black dots) between August 29, 2014 and December 31, 2016 (model settings: sensitive). Panel b shows the match ratio [0–1] between NatCatSERVICE and our events detected at the level of admin 1 subdivisions aggregated per country. The labels show the total number of flood events recorded in NatCatSERVICE between August 29, 2014 and December 31, 2016 (model settings: sensitive).

Figure 4a shows the number of events detected by our algorithm at admin 1 level, either aggregated by country or shown at admin 1 level, as well as the events recorded in Munich Re’s NatCatSERVICE. Due to the relatively short overlap between the databases the available data in NatCatSERVICE (1960–2016) and our detected events, the spin-up period is included. The data thus ranges from July 30, 2014, until December 31, 2016. Within this period, NatCatSERVICE records 1260 flood events in 154 countries, including the date of the event onset, end date, and its location, while we record 9,821 events at admin 1 level using the sensitive run within the same time period. Like other databases such as EM-DAT, the NatCatSERVICE database covers most of the large flood events around the world, but has limited records of small floods and events in developing countries with restricted connectivity^4^.

The methods used in this manuscript based on Social media analytics and the methods for recording events in disaster databases such as NatCatSERVICE are different. For example, most traditional databases often only include flood events based on certain threshold (e.g., the amount of damage and number of people affected). In contrast, people on social media often refer to a flood event even if a small number of streets is inundated. Therefore, online media databases and traditional databases are not directly comparable, but we do note that similar patterns appear (Fig. 4b). Illustrative differences can be seen in areas such as the Philippines and Indonesia, whose first official language is accounted for in our algorithm. Here, we seem to find more events than listed in NatCatSERVICE. By contrast, more events are found by NatCatSERVICE in areas such as China and the Middle East, where languages like Mandarin and Arabic, which are not included in our algorithm, are widely spoken and Twitter penetration is lower.

To further compare our detected events with NatCatSERVICE, we drew a buffer with a radius of 100 km around the main coordinate of each riverine flood, flash flood, or tsunami recorded in NatCatSERVICE between August 29, 2014 and December 31, 2016. Next, we check whether at least one of the events detected by our algorithm intersects the buffer around the NatCatSERVICE’s event within a range of 5 days and analyse the “match ratio” [0–1], defined as the ratio of events in NatCatSERVICE that is also detected by our algorithm. Similar to the previous analysis, the spin-up period is included due to the short overlap between both databases. In Fig. 4b we show the match ratio for events detected at the level of admin 1 subdivisions, aggregated per country. In general, two major patterns emerge from the data: (1) countries for which we included the first official language in our model (Table 1) generally have a higher match ratio and (2) countries with a relatively high Twitter penetration^38^ seem to have a higher match ratio. For example, countries where the first language is included in our study, including most Western countries, South America, the Philippines and Indonesia, show a relatively high match ratio. This may also be accounted for by the fact that these countries have a relatively high Twitter penetration. India, Myanmar and Thailand also show a high match ratio, even though Twitter penetration in these countries is relatively low. This might be explained by the fact that the floods in those countries received a good deal of media attention in the United Kingdom. In the case of India, the fact that English is recognized as an official language may also be a contributing factor.

Table 4 shows that when the impact of flooding is larger in both financial and humanitarian terms (denoted as catastrophe class in NatCatSERVICE), the match ratio increases. This can be expected, since more severe events usually impact more people and receive more news coverage, which increases the likelihood of people and organizations tweeting about an event. The match ratio is slightly lower when only the events detected at an admin 1 subdivision are taken into account, indicating that some events are only detected at the national level. Analysing only events detected at the admin 1 subdivision in countries whose first official language is included in our model results in a higher match ratio, indicating that the algorithm works better in those countries. This is also in line with the observations in Fig. 4b.

**Table 4:** Correlation per catastrophe class between detected events and NatCatSERVICE between July 30, 2014 and December 31, 2016 (model settings: sensitive).

Catastrophe Class*	Number of events in NatCatSERVICE	Match ratio	Match ratio at admin 1	Match ratio at admin 1, official language analysed
0+	1260	0.81	0.55	0.63
1+	772	0.86	0.58	0.68
2+	369	0.92	0.63	0.75
3+	124	0.96	0.73	0.89
4+	19	1.00	0.89	1.00

*No event with class 5 or 6 occurred in the analysed period.

To check for differences in internet use across countries, we calculated the match ratio for different income groups as defined by the World Bank (Table 5). We found the match ratio is slightly lower in low income countries, which can be expected due to an average lower Twitter penetration in those countries.

**Table 5:** Correlation per income class between detected events and NatCatSERVICE between July 30, 2014 and December 31, 2016 (model settings: sensitive).

Income class	Number of events	Match ratio
High income	359	0.90
Upper middle income	401	0.81
Lower middle income	386	0.83
Low income	108	0.74

### Manual validation of found events

Because the majority of floods have remained unreported in the past^4^, no comprehensive database of flood events exists as is the case for earthquakes^39^. Moreover, an exact start and end time of these events is hard to estimate. While this further underscores the value of flood event detection from online media, it hampers the ability to establish both overall exact precision and recall scores using automated analysis. Therefore, we manually validated 100 randomly selected flood events for the three model runs (sensitive, balanced and strict) by reading the tweets (Table 6). Here, we detect an event if the tweets appear to refer to an actual event, including the right time and location, and if people are referring to the event as a flood. In case of doubt, we check additional sources to confirm it is an actual flood event. If still unsure, we do not label it as a flood event. Moreover, we only consider events where flooding appears to be caused by excessive water from natural sources (e.g., we include dam breaches but not pipe bursts from the water supply). The most important reasons floods detected by our algorithm were not actual flood events are (1) wrong location assigned, (2) another topic (e.g., “increasing floods due to climate change” and “streets are flooding with protestors”), (3) bursts of water pipes and (4) tweets relating to a historic disaster.

**Table 6:** Number of detected events, their precision scores and the estimated number of true positive events for the three model runs between July 30, 2015 and November 20, 2018.

	Sensitive (c = 1)	Balanced (c = 2)	Strict (c = 3)
Number of events	13,931	10,551	8,588
Precision (established through manual validation of 100 events)	0.76	0.87	0.90
Estimated number of true positive events (correct events)	10,600	9,200	7,700

Table 6 shows the effect of changing parameter *c* on the precision (equation 1) of the algorithm. As expected, when the constants *c* is lowered, more events are found by the algorithm. In the strict run (*c* = *3*), we found the highest percentage of correct events (90%), while the sensitive run (*c* = *1*) had the lowest percentage of correctly detected events (76%). Because the total number of actual flood events is unknown, exact recall (equation 2) measures cannot be established. However, we can analyse the relative change between runs by estimating the total number of correct events at around 10,600 for the sensitive run and approximately 7,700 for the strict run. Since the number of actual events is the same in both cases, we can derive that recall is approximately 37% higher in the most sensitive run (*c* = *1*) comparted to the strict run (*c* = *3*).

Finally, we analysed events from the *sensitive* run that were not registered in NatCatSERVICE between the end of the spin-up period (July 30, 3015) and the last available events in NatCatSERVICE (December 31, 2015). To do so, we created a 100 km buffer and 5-day time window around each event coordinate in NatCatSERVICE, similar to the methodology used to establish the match ratio. Then, we selected the events that did not intersect any buffer, both in space and time. We found that out of 1,769 events detected using our algorithm during this period, 1,168 were not available in NatCatSERVICE (~66%). From these events that were unregistered in NatCatSERVICE, we randomly selected 100 events for manual validation and found that 62% of them was an actual event. This is in line with previous observations that the majority of floods are undetected and are therefore not included in disaster databases^4^. This shows that social media can be a valuable complementary source for monitoring flood events.

## Usage Notes

Our Twitter algorithm runs in real-time and all data obtained (historic and real-time) is published on a freely accessible web platform (www.globalfloodmonitor.org), and can be immediately applied to a wide range of applications. Our algorithm finds a higher number of flood events available within a much shorter time frame than traditional disaster databases and can be used to serve several applications, including:

- The validation of flood forecasting models. Several flood forecasting models are currently in use or being developed. However, these forecasting models require large amounts of data to both validate and calibrate the models. This data continues to remain scarce^40,41^.
- As the output of the described algorithm is available in real-time, it could be used for data assimilation in flood prediction models.
- The tasking of satellites. During natural hazards, satellites can be tasked (i.e., pointing satellites to an area of interest) to obtain more frequent imagery of the impacted area to improve event extent detection, disaster response and recovery^42,43^. As our algorithm operates in real time, it allows for rapid identification of events and the immediate tasking of satellites.
- The use of instantly detected events for disaster response agencies. While detection on admin 1 level is relatively coarse for most countries, the relevant tweets can be easily reviewed by response agencies to obtain more detailed information.
- The support of applications that depend on historical flood information, such as forecast based financing schemes^22,44^.

We should note that social media data is not evenly distributed over the globe and is heavily dependent on a variety of factors including population density, socio-economic development, access to the internet, and regional preferences for specific platforms. Additionally, Twitter usage is not evenly distributed over time. The platform’s popularity varies over time and user activity is not evenly distributed throughout the day. Therefore, this method should be considered as a complementary method for detecting, and providing information on floods, alongside other methods, each hampered with their own shortcomings.

## Data Availability

The datasets generated have been created using code for Python 3.6, Elasticsearch 6.6 and PostgreSQL 10.6. The code is available through https://github.com/jensdebruijn/Global-Flood-Monitor.
